# Elective use of surgical cricothyroidotomy for maxillofacial fracture fixation with contraindication of nasotracheal intubation: a case report

**DOI:** 10.1186/s40981-015-0021-6

**Published:** 2015-10-16

**Authors:** Masayuki Kuroiwa, Kenichi Kumazawa, Sohei Ito, Masayasu Arai, Hirotsugu Okamoto

**Affiliations:** 1 Department of Anesthesiology, Kitasato University, School of Medicine, 1-15-1, Kitasato, Minamiku, Sagamihara, Kanagawa Japan 2520374; 2 Department of Emergency and Disaster Medicine, Kitasato University, School of Medicine, Sagamihara, Kanagawa Japan; 3 Research and Development Center for New Medical Frontiers, Kitasato University, School of Medicine, Sagamihara, Kanagawa Japan

**Keywords:** Surgical cricothyroidotomy, Maxillofacial surgery, Tracheostomy, Subglottic stenosis

## Abstract

We report three cases of airway management with elective surgical cricothyroidotomy (SCT) for anesthetic management during surgical repair of maxillofacial injury involving basal skull fracture or nasal-bone fracture. In all patients, general anesthesia was induced, a supraglottic airway (SGA) device inserted, and SCT performed. Tracheal intubation was performed through SCT site, and the SGA device was removed. After surgery of maxillofacial fixation, the SGA device was re-inserted and the tracheal tube was removed. No major complications, such as subglottic stenosis or voice change, occurred. SCT holds potential as an alternative to tracheostomy because of ease of performance, fewer complications, and better cosmetic outcomes.

**Electronic supplementary material:**

The online version of this article (doi:10.1186/s40981-015-0021-6) contains supplementary material, which is available to authorized users.

## Background

Surgical cricothyroidotomy (SCT) has become known as a suitable procedure for emergency surgical airway management, and elective use of SCT is currently rare. In anesthetic management for open reduction and fixation surgery (ORF) of maxillofacial fracture with intermaxillary fixation, the nasotracheal intubation technique is often used, as oral intubation may need to be avoided for particular reasons. For instance, the surgeon may require intermaxillary fixation to inspect fracture reduction and maintenance of the reduction during surgery, and it is not desirable to fumble with a tracheal tube during surgery that requires an intraoral approach. However, nasotracheal intubation may be contraindicated in patients simultaneously suffering from a skull-base fracture, particularly those with cerebrospinal fluid rhinorrhea, because of the increased rate of intracranial infection and the fact that nasotracheal intubation may aggravate the trauma . In such a situation, tracheostomy is often selected for ORF of the maxillofacial fracture with intermaxillary fixation in patients with a skull-base fracture. However, tracheostomy may have certain disadvantages, such as serious complications (8–24 %) [1] and tracheal stenosis (1–20 %) [2]. In contrast, SCT is easier, quicker, and safer to perform than tracheostomy [3], but few studies have investigated the usefulness of SCT for temporary airway management in maxillofacial bone surgery [4–6].

Here, we report three cases in which airway management was achieved with elective SCT for anesthetic management, without major complications, during surgical repair of maxillofacial injury with a skull-base fracture or a nasal-bone fracture.

### Anesthetic management with SCT for maxillofacial surgery

**Fig. 1:**
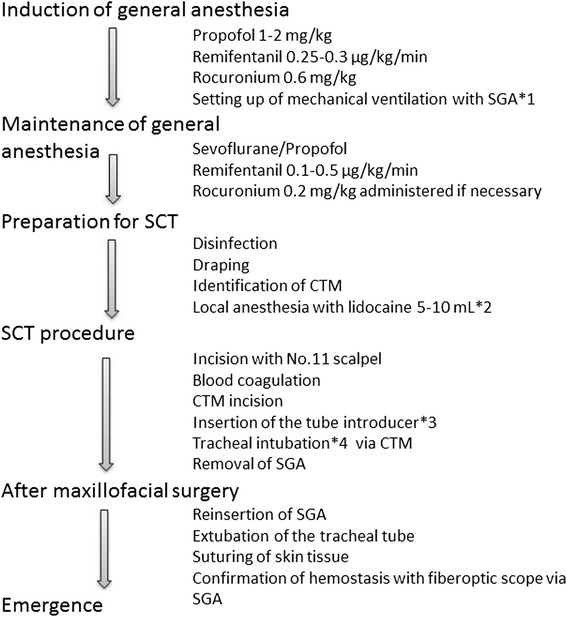
Anesthetic management for oral maxillofacial surgery with SCT. CTM: cricothyroid membrane, SCT: surgical cricothyroidotomy, SGA: supraglottic airway *1: I-gel® (Intersurgical Ltd., Japan), *2: add adrenaline 10 μg per 1 mL, *3: Frova® intubating introducer (COOK Medical, USA), *4: ID 6.0–6.5 mm Parker Flex-Tip® reinforced tube (Parker Medical, Fairfield, NJ, USA)

**Fig. 2:**
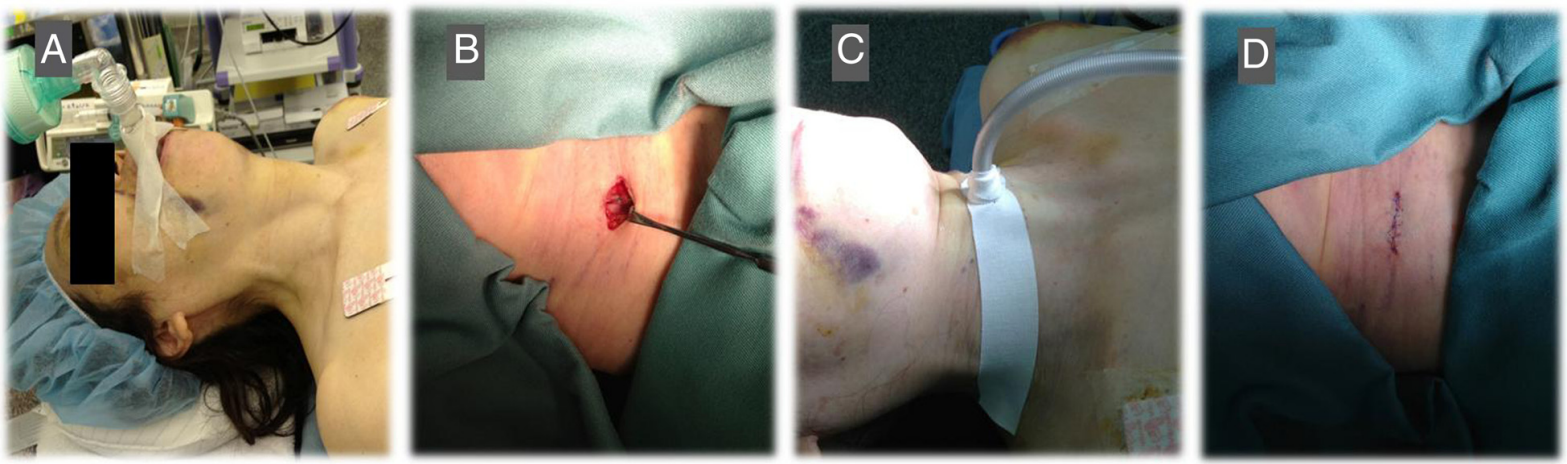
Typical general anesthetic management with SCT (pictures from Case 3). **a**: General anesthesia was induced with SGA device insertion. **b**: The cricothyroid muscle was dissected and the CTM exposed. **c**: The tracheal tube was secured during surgery. **d**: The SGA tube was reinserted after surgery, and the skin incision was sutured after extubation of the tracheal tube from the CTM. CTM: cricothyroid membrane, SCT: surgical cricothyroidotomy, SGA: supraglottic airway

Each patient elected to undergo SCT after being informed about the equipment, method, and advantages and disadvantages of both tracheostomy and SCT for maxillofacial bone surgery, and thereafter provided informed consent. The procedure was performed by the same anesthesiologist with sufficient clinical and practical experience in SCT. All anesthetic management procedures followed the same protocol (Figs. 1 and 2). The clinical characteristics of the three cases are shown in the table.

## Case presentation

### Case 1

**Fig. 3:**
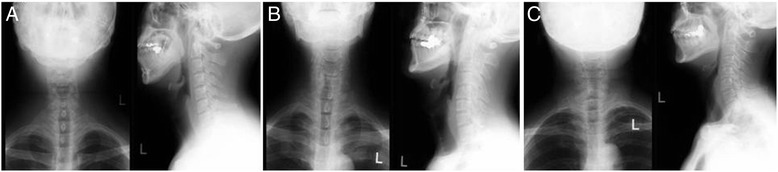
X-ray images of the larynx a few months after surgery. No subglottic stenosis was observed in any of the cases. **a** X-ray image of Case 1 at 5 months after surgery. **b** X-ray image of Case 2 at 4 months after surgery. **c** X-ray image of Case 3 at 7 months after surgery

A 28-year-old man suffered fractures of the mandible and skull-base in a traffic accident involving a bus while cycling. He underwent ORF of the mandible 10 days after the injury. Airway management was achieved using the protocol for SCT depicted in Fig. 1 for SCT during maxillofacial surgery. The SCT was performed using the “four-step technique” as used for emergency airway management. The duration of SCT after induction of general anesthesia was 8 min. After completion of maxillofacial surgery, the tracheal tube was removed from the SCT and the I-gel® (Intersurgical Ltd., Tokyo, Japan) reinserted before the patient recovered. Subsequently, minor bleeding occurred from the branches of the anterior jugular vein in the SCT and blood entered the trachea. The surgeon then achieved hemostasis by electrocoagulation. After the SCT site was sutured with 3–0 nylon, the patient awoke fully without any complications. The patient progressed well postoperatively and was discharged 16 days after surgery. At the 5-month follow-up, no adverse symptoms, such as voice change or discomfort, were reported and the surgical scar at the SCT site was already fading (Additional file 1: Figure S1); subglottic stenosis of the larynx was not observed on radiography (Fig. 3a).

### Case 2

A 47-year-old man sustained extensive maxillofacial trauma, including basal skull fracture. ORF of the maxillofacial bones with elective SCT was performed 9 days after the injury. In this patient, a deformation of the larynx due to a childhood injury made it difficult to identify the cricothyroid membrane (CTM) accurately. At the 4-month follow-up after surgery, the surgical scar at the SCT site was inconspicuous and subglottic stenosis of the larynx was not observed on radiography (Fig. 3b).

### Case 3

A 62-year-old woman sustained multiple maxillofacial trauma injuries and nasal-bone fracture after being hit by a truck while walking. Her elective SCT was performed using the same procedure as above. The duration of SCT was 12 min. When the patient was discharged on postoperative day 7, she was reportedly very satisfied with the small size of the skin incision. No complication of SCT was observed at the 8-month follow-up (Fig. 3c).

## Discussion

We report three cases in which anesthetic management was achieved with elective SCT, without major complications, during maxillofacial surgery. Tracheostomy is often selected over SCT in similar cases because of concerns about subglottic stenosis [6], largely owing to a paper published by Jackson in 1921 [6, 7], which warned of a high incidence of subglottic stenosis after high tracheostomy (cricothyroidotomy), in a study based on 158 cases. In addition, other reports have been published supporting the theory about increased risk of subglottic stenosis with SCT [8–10]. However, Brantigan and Grow [11] reported that no subglottic stenosis was observed in 655 cases in which they performed elective SCT, and Holst et al. [12] found no severe perioperative or postoperative complications in their 103 elective SCT cases. Recently, Teo et al. [6] reported that the incidence of subglottic stenosis was very low (0.5–0.7 %), after reviewing 1916 cases of SCT, and thus recommend the use of elective SCT rather than tracheotomy in cases where intermaxillary fixation is required.

Other complications of SCT, besides subglottic stenosis, are major bleeding and voice changes [5, 6, 13]. Brantigan et al. [11] reported an incidence of intraoperative or postoperative bleeding of 1.5 % (10/664) in elective SCT cases, although no case required transfusion. However, if the tracheal tube is removed from the CTM after a short period of time, bleeding may reoccur from the incision. In fact, we observed blood entering the trachea from the CTM in Case 1. In subsequent cases reported here, we were able to identify the branches of the anterior jugular vein in the incision site. In cases in which the SCT tube will remain in place for a short period of time, we recommend applying hemostasis by ligation if these vessels are observed.

Voice change was indicated by Holst et al. [13] as one of the main complications. Brantigan et al. [11] observed severe voice changes in 1.1 % (7/664) of elective SCT cases. Voice change may occur due to dysfunction of the cricothyroid muscles. Therefore, disruption of the cricothyroid muscle during dissection of the CTM should be avoided. On the other hand, change in voice quality is not a specific complication of SCT. Rehm et al. [14] examined the incidence of voice change with both SCT and tracheostomy in trauma patients, and found no significant difference between the two groups.

The noteworthy points of our experience are as follows. First, the elective SCT technique should differ from that for emergency airway management. In Case 1, we followed the four-step technique, and bleeding was observed from the branches of the anterior jugular vein after the tracheal tube was removed from the CTM postoperatively. Elective SCT does not need to be performed urgently; thus, adequate hemostasis is recommended. In fact, the branch of the anterior jugular vein was identified in the site of the SCT in all our cases (Table 1). Second, if the larynx is displaced anatomically, SCT should be performed in the presence of a laryngeal specialist, such as an otolaryngologist or a cervical surgeon. In Case 2, the larynx had shifted to the right because of trauma sustained by the patient in childhood. Finally, in Case 3, the patient chose to avoid tracheostomy mainly for cosmetic reasons. In SCT, the incision is located higher, but is shorter, than that in tracheostomy. Therefore, cutting along a skin secant may yield a better cosmetic outcome.

**Table 1:** Clinical characteristics of three patients with elective surgical cricothyroidotomy

	Case 1	Case 2	Case 3
Age (y.o.)/Gender	28/m	47/m	62/F
ASA-PS	II	II	I
Major trauma	Mandbular Fx.	Maxilb facial Fx.	Maxilb facial Fx.
Basal skull fracture	+	+	-
Nasalbone fracture	-	-	+
Duration from injury to surgery (day)	10	7	8
Size of SGA	4	4	3
D of intubated tube (mm)	65	60	60
Duration of SCT (min)^*1^	8	21	12
Duration of surgery (min)	150	255	160
Duration of anesthesia (min)	330	300	320
Intemaxillary fixation	+	+	+
Airway management at emergence	SGA	SGA	SGA
Issures with SCT procedure	Minor bleeding	Shifted anatomy	-
Duration of follow-up (month)	5	4	8
Long term complication with SCT^*2^	-	-	-

*SCT* surgical cricothyroidotomy, *SGA* supraglottic airway, *Fx* fracture

^*1^duration from establishment mechanical ventilation with SGA to finishing tracheal intubation via CTM

^*2^subglottic stenosis, voice change, dysphagia were assessed

## Conclusion

We here reported three cases of airway management with elective SCT for anesthetic management in ORF of maxillofacial injuries involving basal skull fracture or nasal-bone fracture. SCT holds potential as an alternative technique to tracheostomy, because it is easy to perform, has fewer complications, and has better cosmetic outcomes.

## Consent

Written informed consent was obtained from the patients for publication of this case report and accompanying images. A copy of the written consent is available for review by the Editor-in-Chief of this journal on request.

## Additional file

Additional file 1: Figure S1. The surgery scar at the surgical cricothyroidotomy site at 5 months after surgery. The scar is inconspicuous (white arrow). (JPEG 49 kb)

## Abbreviations

CTM: Cricothyroid membrane
ORF: Open reduction and fixation surgery
SCT: Surgical cricothyroidotomy
SGA: Supraglottic airway
