# Supplementary figures and images for: Elective use of surgical cricothyroidotomy for maxillofacial fracture fixation with contraindication of nasotracheal intubation: a case report
Source: JA Clin Rep. 2015 Oct 16;1(1):16. doi: 10.1186/s40981-015-0021-6 (PMC5818707; doi:10.1186/s40981-015-0021-6)

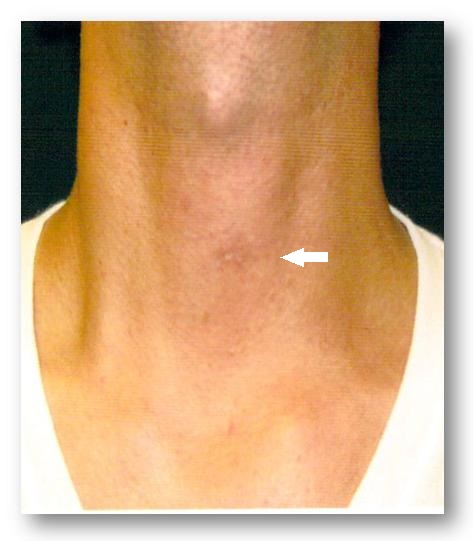

Supplement: Supplementary file 1 — The surgery scar at the surgical cricothyroidotomy site at 5 months after surgery. The scar is inconspicuous (white arrow). (JPEG 49 kb) [file 40981_2015_21_MOESM1_ESM.jpg]
